# Cytokine and chemokine profile of the innate and adaptive immune response of *schistosoma haematobium* and *plasmodium falciparum* single and co-infected school-aged children from an endemic area of Lambaréné, Gabon

**DOI:** 10.1186/s12936-015-0608-4

**Published:** 2015-02-25

**Authors:** Ulysse Ateba-Ngoa, Ayola Akim Adegnika, Jeannot F Zinsou, Roland F Kassa Kassa, Hermelijn Smits, Marguerite Massinga-Loembe, Benjamin Mordmüller, Peter G Kremsner, Maria Yazdanbakhsh

**Affiliations:** Department of Parasitology, Leiden University Medical Center, Albinusdreef 2, 2333 Leiden, ZA The Netherlands; Institut für Tropenmedizin, Universität Tübingen, Wilhelmstraβe 27, D-72074 Tübingen, Germany; Centre de Recherches Médicales de Lambaréné, BP: 118, Lambaréné, Gabon

**Keywords:** Malaria, Schistosomiasis, Co-infection, Innate immune response, Adaptive immune response, Principal component analysis, Epidemiology, Lambaréné, School-aged children

## Abstract

**Background:**

Helminths and malaria are among the most prevalent infectious diseases in the world. They both occur in tropical area where they often affect the same populations. There are studies suggesting an effect of helminths on malariometric indices. For example, malaria attacks as well as disease severity has been shown to be influenced by a concurrent chronic helminth infection. However, there are also studies that show no effect of concurrent helminth infections on malarial outcomes. To start addressing this issue, the effect of chronic *Schistosoma haematobium* infection on both the innate and adaptive immune response of *Plasmodium falciparum*-infected subjects was assessed in an area endemic for both these infections in Gabon.

**Method:**

Subjects infected with *S. haematobium* and or *P. falciparum,* as well as a control group with neither of these infections, were recruited. For innate immune response, heparinized blood was obtained and cultured for 24 hours with a panel of TLR ligands. For adaptive immune response, PBMC was isolated and stimulated with SEB for 72 hours. Cytokines and chemokines were measured in supernatants using a multiplex beads array immunoassay. Principal Component analysis was used to assess pattern of cytokine and chemokine responses representing the innate and adaptive components of the immune system.

**Results:**

Overall it was observed that the presence of *P. falciparum* infection was marked by an increase in innate and adaptive immune responsiveness while *S. haematobium* infection was characterized by an increased chemokine profile, with at the same time, lower pro inflammatory markers. When the study subjects were split into single infected and co-infected groups no effect of *S. haematobium* on the immune response of *P. falciparum* infected subjects was observed, neither for the innate nor for the adaptive component of the immune response.

**Conclusion:**

This study provides original information on the cellular immune response of *S. haematobium* and/or *P. falciparum* in infected subjects. It rules out an effect of *S. haematobium* on the cytokine profile of subjects co-infected with *P. falciparum*.

## Background

In *Plasmodium spp*. infected subjects the ability to control the development of the parasite depends largely on the balance between pro and anti-inflammatory mediators of their immune response [1,2]. Acute *Plasmodium falciparum* infection is usually associated with an increase of INFγ and TNF, regarded as the markers of the Th1 and pro-inflammatory response [2,3]. This pro-inflammatory response is thought to be needed to impede the multiplication of the parasite and favour its clearance, both in human and animal models [2-4]. While important for parasite clearance a powerful Th1 and pro-inflammatory response could also be detrimental for the host if uncontrolled, leading to tissue damage and severe disease [5,6]. This is supported by the importance of the anti-inflammatory network characterized by an expansion of the regulatory T cells [7-10], as well as by the activation of negative regulators like the CTLA4 or PD-1, transmembrane receptors, during malaria infection [11,12]. Moreover, as Th1 responses can be counteracted by Th2 cells, the presence of a strong Th2 response might also influence anti-malarial immunity.

In areas where malaria is endemic, it is the norm that *Plasmodium-*infected people also suffer from a concurrent helminth infection [13,14]. Helminths have repeatedly been shown to modulate the immune system of their host in order to survive [15]. Chronic helminthiasis is usually characterized by a marked Th2 response [16,17] as well as by the induction of a regulatory network [18,19] that could consequently impair the host immune response to other antigens [20]. Whether a concurrent helminth infection of the host can affect his immune response to *Plasmodium spp.* co-infection is still debated [21,22]. Population-based studies conducted to assess the effect of helminths on malariometric indices and on the immune response of *P. falciparum* infected subjects have so far revealed contrasting results. For example, in Senegal, Sokhna *et al.* observed that children with *Schistosoma mansoni* had an increased incidence of clinical malaria in comparison to their uninfected counterparts [23], while in Mali, Lyke and colleagues reported a protective effect of *S. haematobium* infection against malaria [24]. A similar divergent picture has also emerged when considering the cellular response of malaria and helminth co-infected subjects. For example in Senegal, Diallo *et al.* reported a significant increase of the plasma concentration of TNF and IFNγ measured in *S. haematobium* and *P. falciparum* co-infected children in comparison to their *P. falciparum* single infected counterpart [25]. In the same studies, they also observed a significant increase of the plasma concentration of TNF, IFNγ, IL-10, TGF-β, sTNF-RI and sTNF-RII rates in co-infected subjects [25]. Similarly in Ghana, Hartgers *et al.* compared the cytokine response of *S. haematobium* subjects to uninfected ones when their whole blood were stimulated with *P. falciparum* infected red blood cells (iRBCs) and observed that the measured level of IL-10 was significantly higher in the infected group [26]. Inversely, in Mali Lyke *et al.* reported a decreased level of IL-10 in plasma from *S. haematobium* and malaria co-infected subjects by comparison to malaria only subjects [27].

Some reports have suggested that a concurrent helminth infection is associated with elevated cytokines in particular pro inflammatory ones compared to *P. falciparum* infected subjects [25,26], while in others either no effect or even a decreased in these cytokines [27-29]. It is important to note that each of these studies assessed the immune system of infected people from a different angle, either by using different stimuli or by characterizing a different cells type. Moreover none has yet attempted to provide information on how helminths affect both the innate and the adaptive immune response of *P. falciparum*-infected subjects within the same cohort.

This study provides information on the cellular immune response of *P. falciparum*-infected subjects, with or without concurrent *S. haematobium* infection. Instead of assessing cytokines responses individually, a more global approach was taken to profile the pattern of cytokine responses in the study subjects. The study hypothesis was that a comprehensive and integrative assessment of multiple cytokines involved in the innate or the adaptive immune response of co-infected subjects would provide a better insight into the effect of *S. haematobium* on the immune response of *P. falciparum* infected subjects.

## Methods

### Recruitment of study participants and diagnosis of parasitic infections

This study was cross sectional and was conducted in the Bindo village located in the Moyen-Ogooué province in Gabon. The Bindo village is endemic for both *S. haematobium* and malaria [30]. School children from six to 16 years of age, attending the only school of the village were included. Urine and blood samples were collected at inclusion for the diagnosis of *S. haematobium* and *P. falciparum* infection as well as for immunological assays. A thorough description of the parasitological test and the immunological assays has been previously published in a study protocol article [31]. Briefly, *S. haematobium* infection was determined by the detection of eggs by microscopy in 10 ml of filtrated urine. *Schistosoma haematobium*-uninfected subjects were those who did not show any eggs in three samples of urine collected on three consecutive days. Detection of *P. falciparum* infection was made by real time-PCR performed on DNA extracted from EDTA blood pellet kept frozen in - 80°C [32].

### Immunological assays

Peripheral blood was collected in sodium heparinized tubes for every child as described elsewhere [31]. To assess the innate immune response heparinised blood was diluted in RPMI (1:1) and cultured for 24 hours at 37°C with a panel of five different Toll like receptor ligands (LPS [TLR4], PAM3 [TLR1/2], CPG [TLR9] CL097 [TLR7/8]), *S. haematobium* eggs antigens (SEA) and a combination of LPS and SEA. Supernatant was collected after 24 hours and cytokine production was measured using the multiplex beads array immunoassay. The cytokines/chemokines quantified for this ex-vivo assay were IFNα2, IL-1ß, IL-6, IL-10, IL-12p70, IL-13, IFNγ, MCP-1, MIP-1α, MIP-1ß, TNF and IP-10.

In order to assess the adaptive immune response, PBMC were isolated by density gradient centrifugation on Ficoll as already described [31]. PBMCs were cultured for 72 hours at 37°C with SEB and supernatant was collected for the measurement of a panel of 11 different cytokines (TNF, IFNγ, IL-2, IL-4, IL-5, IL-13, IL-17A, IL-17 F, IL-22, IL-10, and IL-21) by a multiplex beads array immunoassay.

### Statistics

Chi square and fisher’s exact test were used to compare categorical variables. Not normally distributed quantitative variables were transformed either by Log10 or Box-Cox transformation. Student *t*-test and ANOVA were performed when data met the assumption of normality. Otherwise the non-parametric Mann Whitney and Kruskal Wallis tests were used. Correlation between two continuous variables was assessed using the spearman rho test.

Principal component analysis (PCA) was carried out on the cytokine variables in order to summarize them. Of note PCA is a mathematical technique that allows reducing the dimension of large dataset by identifying new summary variables also called principal component (PC). Each principal component is made of a set of original variables that share a certain level of correlation. All the analysis was performed on medium subtracted data. Negative values were set to zero. Cytokine from the innate [obtained after whole blood stimulation] and the adaptive panels [obtained after PBMC stimulation] were analyzed independently by PCA. As mentioned above the innate panel consisted of 12 cytokines measured after stimulation of cells by six different conditions. Before performing the PCA an average cytokine response were calculated for each subject by taking the mean of the cytokine level obtained with the six different stimuli. This step only concerned the innate panel and was not needed for cytokine of the adaptive panel since the cytokine response to one stimulus was only assessed. All variables were transformed either using a log10 or a Box-Cox transformation to reduce skewness prior to PCA. Principal components identified were considered for further analysis if their eigenvalues were above one. No rotation was applied. Individuals PC scores were obtained for each subject and used for comparison between groups. All the statistical tests were computed using R version 3.0.1. The R packages ggplot2 version 0.9.3.1 and FactoMineR version 1.25 were used for making graphs and performing the PCA respectively. Statistical significance was set for p value below 0.05.

### Ethics

The study was approved by the “Comité d’éthique Régional de Lambaréné” (CERIL). Informed consent was obtained from parents or legal guardians of each of the children included in the study. Appropriate treatment was given to children found with *P. falciparum* or *S. haematobium* infection as per the local guidelines.

## Results

### Characteristics of the study subjects

The recruitment of the study participants took place in May 2011. Overall 125 subjects aged from six to 16 years were included. Among them 63 (50.4%) were infected with *S. haematobium* while 66 (53%) carried *P. falciparum* in their blood as determined by PCR. When considering co-infection four different groups were compared as shown in Table 1. Children with *P. falciparum* single infection were younger and had a lower haemoglobin level. No other significant differences were found between the different groups.

**Table 1:** **Characteristics of the study subjects divided by infection status**

	***S.h*** **-/** ***P.f*** **-**	***S.h*** **+/** ***P.f*** **-**	***S.h*** **-/** ***P.f*** **+**	***S.h*** **+/** ***P.f*** **+**	**p-value**
Number of subjects (%)	28 (22%)	31 (25%)	34 (27%)	32 (26%)	-
Gender: M/F	14/14	13/18	19/15	18/14	0.63
Age in years: Median (IQR)	11 (3.25)	11(5)	9.5(4)	13(3)	0.02
Weight in kgs: Median(IQR)	31.5(12.25)	35(15)	30(9.8)	36(18.25)	0.05
Haemoglobin in g/dl: Median (IQR)	11.9(0.8)	12.25(0.95)	11.1(1.08)	11.8(1.6)	0.003
Number of subjects living in the village for more than 5 years (%)	17(61%)	21(75%)	15(44%)	19(61%)	0.10
Number of filaria infected subjects (%)	2(7%)	4(13%)	2(6%)	2(6%)	0.67
Number of subjects previously treated for *S. haematobium*: (%)	9(32%)	21(41%)	7(21%)	14(45%)	0.16
*S. haematobium* eggs count per 10 ml: Median (IQR)	0	15(49.5)	0	52.5(88.5)	0.26^#^
*P. falciparum* CT value Median (IQR)	0	0	28.9 (7.3)	30.7 (8.6)	0.25^#^

*S.h*-/ *P.f*- : Subjects not infected by either *S. haematobium* or *P. falciparum. S.h*+/ *P.f*-: Subjects with single *S. haematobium* infection. *S.h*-/ *P.f*+: Subjects with single *P. falciparum* infection. *S.h*+/ *P.f*+: *S. haematobium* and *P. falciparum* co-infected subjects. M: male and F: female. CT value represents the value of the cycle threshold.

^#^p-value computed to compare infected subjects only.

### Innate immune responses

**Figure 1:**
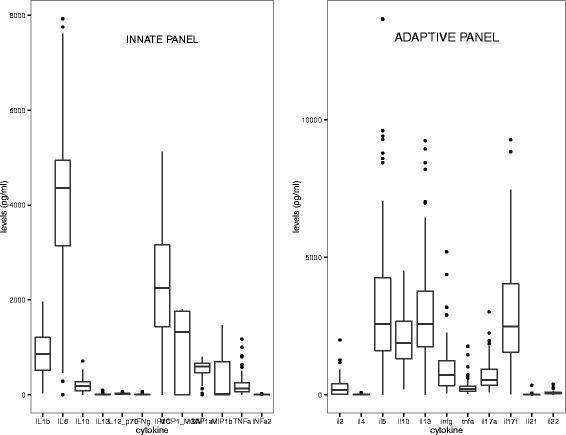
**Levels of the cytokines measured for the innate (left) and the adaptive (right) panels.** For the innate panel the mean response was calculated per cytokine for the 6 different antigens that were used in the whole blood assay. This step was not needed for cytokine pertaining to the adaptive panel since only the cytokine response after SEB stimulation was assessed. Boxes represent the magnitude of the overall response of the study subjects per each cytokine. Whiskers represent minimal and maximal concentrations and dots are indicative of subjects with outlier values.

**Figure 2:**
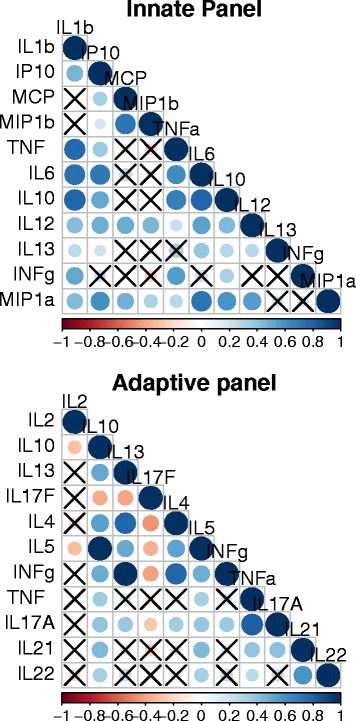
**Correlation matrix of the cytokines of the innate (upper) and the adaptive (lower) panels.** The pair wise correlation between the different cytokines measured is depicted. The intensity of the colours as well as the diameter of the circles give an indication of the degree of correlation between two cytokines and reflect the strength of spearman’s rho correlation coefficient. The crosses represent correlation coefficients that were not statistically significant. Significance was tested using a spearman rank test and level of significance was set at p < 0.05.

Cytokine and chemokines were measured following the stimulation of whole blood with a panel of TLR ligands for 24 hours. In Figure 1a, the levels of measureable chemokines/cytokines are shown. The chemokines/cytokines showed a certain degree of correlation (Figure 2a) that prompted us to perform a principal component analysis. The PCA identified two PCs (respectively named innate PC1 (iPC1) and iPC2) that summarized the 12 measured chemokines/cytokines. These PCs are described in Table 2. Briefly the iPC1 comprised of almost all chemokines/cytokines included in the PC analysis and, therefore, was interpreted as reflecting the general responsiveness. The iPC2 was best characterized by four chemokines/cytokines that clustered into two groups; the MCP1-MCAF/MIP-1β, which were positively loaded and the INFγ/TNF, which were negatively loaded in the PCs. In other words an increase of PC2 would represent an increase of MCP1-MCAF/MIP-1α and a simultaneous decrease of INFγ/TNF.

**Table 2:** **Description of the different principal components identified for the innate panel (iPC1 and iPC2) and the adaptive panel (aPC1, aPC2 and aPC3)**

**Cytokines/Chemokines**	**iPC1**		**iPC2**		**aPC1**		**aPC2**		**aPC3**	
	**Score**	**Contribution**	**Score**	**Contribution**	**Score**	**Contribution**	**Score**	**Contribution**	**Score**	**Contribution**
IL1b	0.4	14	−0.2	6	-	-	-	-	-	-
IP10	0.3	11	-	-	-	-	-	-	-	-
MCP1-MCAF	0.2	2	0.5	27	-	-	-	-	-	-
MIP1a	0.3	12	0.2	5	-	-	-	-	-	-
MIP1b	0.1	1	0.5	29	-	-	-	-	-	-
TNF	0.3	10	−0.4	13	-	-	-	-	-	-
IL6	0.4	17	-	-	-	-	-	-	-	-
IL10	0.4	14	−0.1	2	-	-	-	-	-	-
IL12	0.3	9	0.3	8	-	-	-	-	-	-
IL13	0.2	4	-	-	-	-	-	-	-	-
INFγ	0.2	4	−0.3	10	-	-	-	-	-	-
IL5	-	-	-	-	0.34	11.7	0.55	29.8	−0.13	1.7
IL10	-	-	-	-	0.39	15.1	0.21	4.6	−0.03	0.1
IL13	-	-	-	-	0.35	12.6	0.49	23.7	−0.18	3.4
INFγ	-	-	-	-	0.37	13.7	−0.24	5.6	0.56	31
TNF	-	-	-	-	0.42	17.6	−0.09	0.9	0.49	24.4
IL17A	-	-	-	-	0.36	13.2	−0.31	9.8	−0.13	1.6
IL21	-	-	-	-	−0.31	9.3	0.32	10.8	0.42	17.6
IL22	-	-	-	-	−0.26	6.9	0.38	14.5	0.45	20.23

**Figure 3:**
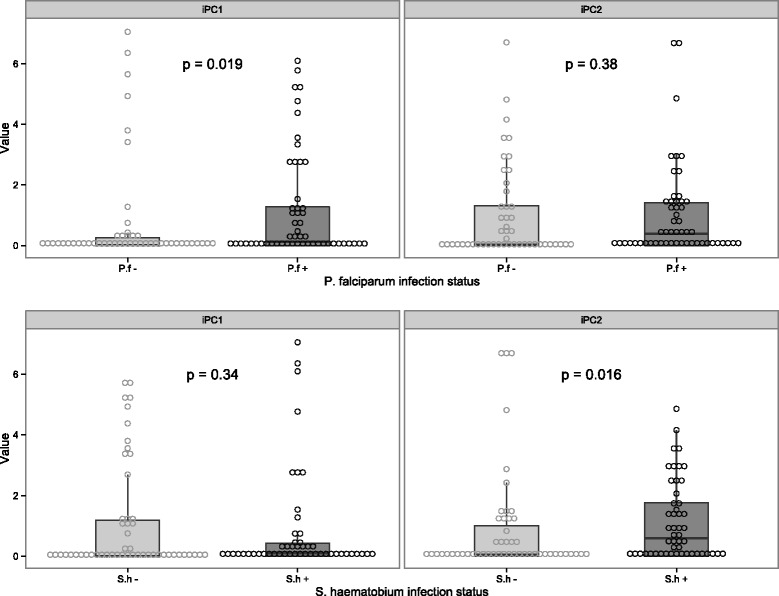
**Effect of** ***P. falciparum*** **and** ***S. haematobium*** **single infection or coinfection on the levels of the principal components reflecting the innate immune response of the study subjects.** Two principal components (iPC1 and iPC2) were identified and explained 67% of the variance in the database. The iPC1 was made of almost all the cytokine included in the model and thus was representative of the innate immune responsiveness of the study subjects. The iPC2, in contrast, was formed by 4 cytokines who clustered into two groups MCP1-MCAF and MIP1b positively loaded in the iPC2 and INFγ and TNF that were negatively loaded. Thus an increase of the iPC2 will mirror an increase of the positively loaded chemokines and a decrease of the negatively loaded cytokines. The box plots represent the median and the interquartile range of the different iPCs while the whiskers show the minimal and maximal value.

Neither age nor gender affected iPC1 or iPC2. In a univariate analysis, iPC1 was higher in *P. falciparum* infected subjects in comparison to subjects with no *P. falciparum* infection (median level of the iPC1 scores: 0.133 in infected *vs* 0.003 in uninfected subjects, p =0.019, Figure 3a), whereas *S. haematobium* infection was associated with an increased iPC2 (0.6 in infected vs 0.04 in uninfected, p = 0.016, Figure 3b). This indicates that during *P. falciparum* infection there is an enhancement of responses to innate stimuli in general while *S. haematobium* infection appears to lead to a selective increase in the release of macrophage-released chemokines, and at the same time to a decrease of pro-inflammatory cytokines in response to TLR stimuli.

To further assess immune response in single and co-infected subjects with particular emphasis on the question whether *S. haematobium* affects response associated with *P. falciparum,* the study population was divided into four groups of uninfected, infected with *P. falciparum* only, *S. haematobium* only and infected with both. What was observed was that iPC1 was higher in those with *P. falciparum* infection, and statistically significantly so in those co-infected with *S. haematobium* (Table 3). These data indicate that *P. falciparum* effect on the immune system is not influenced by concurrent *S. haematobium* infection.

**Table 3:** **Effect of** ***P. falciparum*** **(** ***P.f.*** **) and** ***S. haematobium (S.h)*** **co-infection on the different principal components identified from the innate (iPC) and the adaptive (aPC) immune response**

	***S.h*** **-/** ***P.f*** **-**	***S.h*** **+/** ***P.f*** **-**	***S.h*** **-/** ***P.f*** **+**	***S.h*** **+/** ***P.f*** **+**	**p-value**
	**Median (IQR)**	**Median (IQR)**	**Median (IQR)**	**Median (IQR)**	
iPC1	0 (0–0.03)	0.05 (0–0.31)	0.24 (0–1.28)	0.13(0.005-0.94)	0.046^#^
iPC2	0 (0–0.31)	0.73(0–2.37)	0.12 (0–1.22)	0.53 (0.005-1.48)	0.047^##^
aPC1	0.26 (0.11- 0.89)	0.38 (0.05 – 1.25)	0.36 (0.11 – 1.4)	1 (0.18 – 3.31)	0.22
aPC2	0.53 (0.18 – 0.84)	0.66 (0.2-1.26)	1.27 (0.4-1.7)	0.75 (0.2 -1.6)	0.42
aPC3	0.6 (0.25 – 1.33)	0.47 (0.1-1.6)	1.34 (0.24-3.019)	0.25 (0.05-1.1)	0.56

*S.h*-/ *P.f*- : Subjects not infected by either *S. haematobium* or *P. falciparum. S.h*+/ *P.f*-: Subjects with single *S. haematobium* infection. *S.h*-/ *P.f*+: Subjects with single *P. falciparum* infection. *S.h*+/ *P.f*+: *S. haematobium* and *P. falciparum* co-infected subjects. M: male and F: female. CT value represents the value of the cycle threshold. ^#^Two by two comparison of the groups are shown and show significant difference between *S.h*-/*P.f*- vs *S.h* +/ *P.f* + (p = 0.04). ^##^Two by two comparison show significant difference between *S.h*-/*P.f*- vs *S.h* +/ *P.f* – and between *S.h*-/*P.f*- vs *S.h* +/ *P.f* + (p = 0.03 and 0.02 respectively).

### Adaptive immune responses

As TLRs stimulate the innate immune system in general, to assess the general response of the adaptive immune system the PBMC were stimulated with SEB which is a superantigen capable of triggering a polyclonal T cells activation, part of the general adaptive immune responsiveness. The cytokines measured are shown in Figure 1b and the extent of their correlation in Figure 2b. To profile the cytokine response, a PCA was performed. As shown in Table 2, three different PCs (adaptive PC1 (aPC1), aPC2 and aPC3) were identify. Based on the type of cytokines that contributed to the PC, they were interpreted as follows: aPC1 represented the general immune responsiveness; aPC2 the Th2/Th17; and aPC3 Th1/Th17 response.

**Figure 4:**
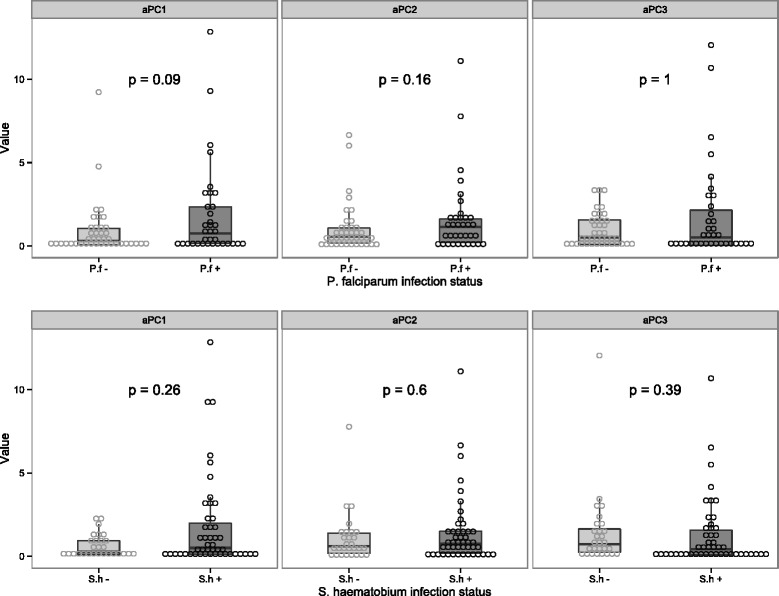
**Effect of** ***P. falciparum*** **and** ***S. haematobium*** **single infection or coinfection on the levels of the principal components reflecting the adaptive immune response of the study subjects.** Three different principal components were identified and explained 76% of the variance in the dataset. The aPC1 was formed by almost all the cytokines included in the model and thus was representative of the adaptive immune responsiveness of the study subjects. The aPC2 and aPC3 was representative of the Th2/Th17 and Th1/Th17 respectively. They were all positively loaded on their respective PCs. The box plots represent the median and the interquartile range of the different iPCs while the whiskers show the minimal and maximal value.

There were no differences between males and females for the aPC1, aPC2, and aPC3 (data not shown). A strong correlation between aPC1 and the age of the study participants (rho = 0.3, p < 0.008) was observed but no effect of age was seen on aPC2 (rho = 0.1, p = 0.3) or aPC3 (rho = −0.1, p = 0.4). As shown in Figure 4a, a trend toward an increase of the aPC1 was seen with *P. falciparum* infection but no effect was observed on the aPC2 or aPC3. Moreover none of the components, aPC1, aPC2 or aPC3 was affected by *S. haematobium* infection (Figure 4b). Finally, no statically significant differences between groups were detected when *P. falciparum* and *S. haematobium* co-infected subjects were compared with those with single or no infection (Table 3).

## Discussion

The main objective of this study was to determine whether chronic *S. haematobium* infection was able to affect the cellular immune response of *P. falciparum* infected subjects. By measuring the cytokine production after in-vitro stimulation, the innate and adaptive immune responses of the study subjects were profiled. Here, rather than assessing single cytokines, the pattern of cytokine responses of the study subjects using a PCA was evaluated. PCA is a mathematical tool widely used in the field of biology. It has the advantage of summarizing highly correlated variables in new latent and synthetic variables called principal components that can unveil new pattern of responses [33,34]. Two PCs (iPCs) that summarize the innate cytokine responses of the study participants were identify as well as 3 PCs (aPCs) for the adaptive cytokine responses. The interpretation of these different PCs shows that cytokines are released with a certain degree of correlation. This is supported by the fact that none of the PCs identified was made of only one cytokine and at least two cytokines were represented in every PC. Moreover, it was noticed that within the same PC cytokines were either negatively or positively correlated. For example in the iPC2, Th1 type cytokines (IFNγ and TNF) were negatively correlated with cytokines released by macrophages (MCP1-MCAF and MIP1β) implying an antagonistic effect that may need further investigations.

In a number of studies it has been shown that *S. haematobium* infection can influence the innate immune response of the human host. For instance in population based studies, schistosomiasis has been linked with functional impairment of human myeloid dendritic cells [35] and their response to TLR ligands [36-38]. *Schistosoma haematobium* excretory-secretory products can prime dendritic cells to shape the adaptive response toward a Th2 phenotype [38,39]. While this immune profile is thought to limit the damage caused by schistosomes in the human host, it could alter the host immune response to a concurrent *P. falciparum* co-infection. What was observed in this study is that *P. falciparum* infection was marked by an increase of the iPC1 and aPC1, which represented the innate and adaptive general immune responsiveness. Interestingly, this was not the case for *S. haematobium* infection, which was associated with an increased level of chemokines (MCP1-MCAF and MIP1b) and the decrease of pro-inflammatory cytokines, namely INFγ and TNF. This indicates that the immune system responds differently to *P. falciparum* and to *S. haematobium* infection. In *P. falciparum*-infected subjects the increase of the iPC1 and aPC1 component is in line with the immune profile seen in asymptomatic *P. falciparum* infected subjects [40]. This is also in line with the literature indicating that in subjects chronically infected with *S. haematobium* there is a down modulation of the pro inflammatory response that is thought to allow the survival of the parasites [18,19]. These observations regarding *S. haematobium* are in line with results of two independent studies that assessed the innate immune response of schistosome-infected subjects. In the first study, Turner *et al.* observed that upon stimulation of whole blood with schistosome excretory/secretory products, *S. haematobium* infected subjects had an enhanced production of IL-10, an anti inflammatory cytokine, whereas the level of the pro-inflammatory cytokine TNF was not different from the uninfected subjects [41]. In the second study, Van der Kleij *et al.* observed that *S. haematobium* infection was associated with a significant decrease in responsiveness to LPS irrespective of pro or anti inflammatory cytokines [37]. However, a study by Meurs *et al.* reported that PBMC of *S. haematobium* infected subjects produced significantly more TNF after stimulation with Pam3 a TLR2/1 ligand in comparison to their *S. haematobium* uninfected counterparts [36]. These differences are difficult to reconcile but the culture methods [whole blood versus PBMC], seasonal fluctuation in immune responses, or other factors such as different environments or co infections [42,43], need to be taken into consideration when comparing studies.

This study did not observe an effect of *S. haematobium* on the innate and adaptive cytokine profile of *P. falciparum* infected subjects. The current body of evidence on helminth and malaria co-infection and its effect on the host immune response has so far given contrasting results. For example a cross sectional study showed no impact of light intensity Ascaris infection on the immune response of malaria infected subjects [44]. In a study conducted in Mali, *S. haematobium* infected and uninfected subjects were followed up until the time to the first malaria episode and serum cytokines were measured at the time of inclusion and at the time when study subjects became infected with *P. falciparum* [27]. At baseline the level of IL-4, IL-6, IL-10 and IFNγ cytokines were all higher in subjects infected with *S. haematobium* by comparison to uninfected subjects. However, when these participants developed an acute episode of malaria, IL-6 and IL-10 cytokines increased considerably in all groups, but to a higher extent in subjects who were free of schistosome infection [27], which would suggest that *S. haematobium* impedes the cytokine storm. It has to also be noted that, looking at the results differently, which is that at the time of malaria infection, the baseline differences in IL-6 and IL-10 in the *S. haematobium* infected and uninfected subjects, fell short of statistical significance, one might conclude that there is no difference between subjects with single malaria versus those who were coinfected. In contrast, in a study in Senegal, where *P. falciparum* infected participants were compared to *S. haematobium* and *P. falciparum* co-infected subjects; Diallo *et al.* reported that the plasma concentration of IL-10, TGFβ, INFγ (but not INFα) was higher in co-infected subjects than in those with single infection. The same authors, when examining *in vitro* production of cytokines by mononuclear cells stimulated with *P. falciparum* schizont extracts and MSP1-19 antigens reported an increase of IL-10 and INFγ but not TGFβ, IL-12 or IL-13 in subjects with *P. falciparum* infection compared with subjects co-infected with *P. falciparum* and *S. haematobium* [45]. Finally, a study conducted by Hartgers *et al.* in Ghana showed higher response to malaria antigens in terms of IL-10 but not INFγ, IL-6, TNF in helminth infected subjects in comparison to those free of helminth infection [26]. It is important to emphasize that in the study of Hartgers *et al.,* the response to malaria antigens was compared between *S. haematobium*-infected and uninfected subjects and, therefore, malaria infection was artificially mimicked by the use of antigens from *P. falciparum*. Regarding, the Senegal studies, the *P. falciparum* singly infected individuals originated from a village where *S. haematobium* infection was never reported before, whereas co-infected subjects were from an entirely different village endemic for both *S. haematobium* and *P. falciparum.* Therefore, it is possible that the differences reported, mirror the exposure to different environmental factors rather than to *S. haematobium*. This is supported by the work by Smolen and colleagues who compared the immune response of children across four different continents. Using a standardized procedure they observed considerable heterogeneity in the cytokine responses in the different geographical areas [42].

One obvious limitation of the present study is that it is cross sectional and one could argue that it does not provide information on history of past helminth infections that are capable of imprinting the host immune system. For example, the innate immune system has been shown to be able to keep a “memory” of early exposure to PAMPs through a process called “trained immunity” which is not addressed in this study [46]. Additional limitation concerns the sample size of the study that may not be sufficient to detect an effect of helminths on *P. falciparum* modulated immune responses. However, this study was carried out in a relatively small community where it was possible to enroll all the school-aged children willing to participate and fulfilling the inclusion criteria. Despite these limitations the present study provides original information on the cellular immune response of *S. haematobium* and/or *P. falciparum* infected subjects. It showed that *P. falciparum,* but not *S. haematobium,* infection was associated with an increase of the immune responsiveness of the study subjects but it did not evidenced an effect of *S. haematobium* on the immune response that were measured in the *P. falciparum*-infected participants.

## Conclusions

This study assessed the effect of *S. haematobium* on the pattern of cytokine responses elicited in subjects concurrently infected with *P. falciparum*. It shows that *P. falciparum* infection is associated with an increased immune responsiveness which is not affected by *S. haematobium* co-infection.
